# Nosocomial Pneumonia in Georgia: A Study of Extended Spectrum Beta-Lactamase (ESBL)-Producing Versus Non-extended ESBL Gram-Negative Bacterial Profiles

**DOI:** 10.7759/cureus.75458

**Published:** 2024-12-10

**Authors:** Giorgi Mgeladze, Giorgi Akhvlediani, Shorena Khetsuriani, Giorgi Maisuradze, Shota Mrelashvili, Vakhtang Robakidze, Ani Papiashvili

**Affiliations:** 1 Microbiology, Tbilisi State Medical University, Tbilisi, GEO; 2 Biomedical Sciences, Georgian American University (GAU), Tbilisi, GEO; 3 Pulmonary and Critical Care Medicine, Tbilisi State Medical University, Tbilisi, GEO

**Keywords:** antibiotic resistance (abr), extended spectrum beta-lactamase (esbl), georgia, gram-negative bacteria (gnb), nosocomial pneumonia

## Abstract

Background: Nosocomial pneumonia is a significant healthcare challenge, particularly in the face of rising antimicrobial resistance among Gram-negative bacteria. The production of extended spectrum beta-lactamase (ESBL) exacerbates treatment complexities.

Aim: This study investigates the prevalence and resistance patterns of ESBL-producing and non-ESBL Gram-negative bacteria in nosocomial pneumonia cases in Georgian hospitals to inform antibiotic stewardship and treatment strategies. To our knowledge, this is the first study of its kind conducted in Georgia, offering critical insights into bacterial resistance in this region.

Methods: This prospective observational study analyzed a total of 357 pulmonary samples from patients diagnosed with nosocomial pneumonia in Georgian hospitals between December 2022 and February 2024. Gram-negative bacterial identification and ESBL determination were performed using the combination disk method, adhering to European Committee on Antimicrobial Susceptibility Testing (EUCAST) standards. The analyses were conducted at TEST-IMP Laboratory and the Richard Lugar Center for Public Health Research to investigate the prevalence and resistance patterns of ESBL-producing versus non-ESBL Gram-negative bacteria.

Results: Among the 256 Gram-negative isolates, 201 (78.5%) were ESBL producers. Pseudomonas aeruginosa (63.7%), Acinetobacter baumannii (18.4%), and Klebsiella pneumoniae (17.9%) were the most prevalent. Non-ESBL producers accounted for 21.5% but exhibited notable beta-lactamase activity. The remaining 101 samples were evaluated as an additional analysis, revealing the distribution of Gram-positive bacteria and fungi as outlined in the results. However, the primary emphasis of this study remains on the resistance patterns and prevalence of Gram-negative pathogens.

Conclusions: The study highlights a concerning prevalence of ESBL-producing bacteria in nosocomial pneumonia cases, emphasizing the urgent need for improved antibiotic stewardship and infection control practices in Georgian hospitals. Non-ESBL producers displayed susceptibility to advanced antibiotics, presenting potential therapeutic opportunities, though vigilance is required to prevent further resistance development.

## Introduction

Nosocomial pneumonia, also known as hospital-acquired pneumonia, is a significant healthcare concern worldwide, accounting for high rates of morbidity and mortality among hospitalized patients [1]. It is defined as pneumonia acquired 48 hours or more after hospital admission, excluding infections present during admission [2,3]. This condition imposes a substantial burden on healthcare systems due to prolonged hospital stays, increased medical costs, and poor patient outcomes. Complications such as sepsis, respiratory failure, and multi-organ dysfunction are not uncommon [1].

Several risk factors contribute to the development of nosocomial pneumonia, including advanced age, prolonged mechanical ventilation, impaired immunity, chronic pulmonary diseases, and prior antibiotic use [4]. Environmental factors, such as hospital crowding and inadequate infection control practices, also play a role in its spread. The condition is further exacerbated by the presence of multidrug-resistant (MDR) pathogens, which significantly reduce therapeutic options [5].

In Georgia, the emergence of extended-spectrum beta-lactamase (ESBL)-producing Gram-negative bacteria capable of hydrolyzing antibiotics such as penicillins and cephalosporins has become a critical public health challenge [6]. These organisms complicate the treatment of nosocomial pneumonia and heighten the risk of resistance proliferation, necessitating urgent action in antimicrobial stewardship [7].

This study investigates the prevalence and resistance patterns of ESBL-producing and non-ESBL Gram-negative bacteria in nosocomial pneumonia cases across Georgian hospitals. By analyzing 357 pulmonary samples collected from three private community hospitals located in the three major cities of Georgia, this study provides critical insights into the evolving landscape of antimicrobial resistance, informing strategies for infection control and improving therapeutic approaches.

## Materials and methods

Sample collection and selection criteria

Sample collection: Samples were collected between December 2022 and February 2024 as part of a prospective observational study conducted across three private community hospitals in Georgia. These specimens were derived from patients' pulmonary samples, including sputum and bronchoscopy materials. After patients gargled with normal saline three times, lower respiratory tract secretions were collected using either a sterile sputum collector or a fiberoptic bronchoscope and placed into a sterile container. Clinically significant values for quantitative culture were defined as <15 squamous epithelial cells/LPF and >20 polymorphonuclear leukocytes/LPF.

Microbiological analysis: Drug sensitivity was determined using the disk diffusion method following the European Committee on Antimicrobial Susceptibility Testing (EUCAST) guidelines [6]. Bacterial isolates from the same patient were considered to be from the same strain.

Inclusion criteria: Adults aged 18 and above diagnosed with nosocomial pneumonia were included if the diagnosis was confirmed by radiological evidence (new or progressive infiltrates) and microbiological evidence (positive cultures from respiratory specimens), with onset occurring 48 hours or more after hospital admission.

Exclusion criteria: Patients with community-acquired pneumonia, prior antibiotic treatment within 14 days, severe immunosuppression, chronic lung diseases, or concurrent infections were excluded from the study [6,8].

Antimicrobial susceptibility testing

Antimicrobial susceptibility testing was conducted on freshly cultured Pseudomonas aeruginosa, Acinetobacter baumannii, and Klebsiella pneumoniae isolates using the Kirby-Bauer disk diffusion method, adhering to the EUCAST guidelines [6]. Bacterial suspensions were standardized to a 0.5 McFarland turbidity, corresponding to approximately 1.5 × 10⁸ colony forming unit (CFU)/mL. These suspensions were uniformly inoculated onto Mueller-Hinton agar plates. Antibiotic-impregnated disks were then placed on the agar surface, and plates were incubated at 37°C for 18-24 hours. Post-incubation, inhibition zone diameters were measured and interpreted as "susceptible," "susceptible, increased exposure," or "resistant" based on the latest EUCAST breakpoint tables (version 14.0). The antibiotics tested included imipenem (10 µg), meropenem (10 µg), ertapenem (10 µg), amikacin (30 µg), gentamicin (10 µg), piperacillin/tazobactam (36 µg), nitrofurantoin (100 µg), trimethoprim/sulfamethoxazole (1.25/23.75 µg), levofloxacin (5 µg), cefepime (30 µg), cefotaxime (5 µg), cefuroxime (30 µg), cefazolin (30 µg), ceftazidime (10 µg), ampicillin/sulbactam (10/10 µg), and ampicillin (10 µg). Isolates exhibiting resistance to at least one agent in three or more antimicrobial classes were classified as MDR [9].

Detection of ESBL-producing strains

For the detection of ESBL production, isolates were subjected to the combination disk test (CDT) as per EUCAST recommendations. Disks containing cefotaxime (5 µg) and ceftazidime (10 µg), both alone and in combination with clavulanic acid (10 µg), were placed on Mueller-Hinton agar inoculated with the test organism. Following incubation at 37°C for 18-24 hours, an increase of ≥5 mm in the inhibition zone diameter for the combination disk compared to the cephalosporin alone indicated ESBL production [6].

Antimicrobial resistance testing of Gram-positive bacteria

Methicillin-resistant Staphylococcus aureus (MRSA) detection was performed according to EUCAST guidelines using cefoxitin (30 µg) disks. Following incubation on Mueller-Hinton agar at 37°C for 18-24 hours, isolates were categorized based on inhibition zone diameters, with resistance indicating MRSA. Oxacillin resistance testing was also conducted to confirm MRSA phenotypes [6].

Induced clindamycin resistance was evaluated using the D-test, wherein erythromycin (15 µg) and clindamycin (2 µg) disks were placed 15-26 mm apart on Mueller-Hinton agar. Isolates exhibiting flattening of the clindamycin inhibition zone adjacent to the erythromycin disk (D-shaped zone) were classified as having inducible clindamycin resistance [6].

The resistance profile of Streptococcus pneumoniae was analyzed against penicillin, cefotaxime, and erythromycin. A significant proportion of isolates exhibited resistance to penicillin, with some categorized as intermediately resistant based on EUCAST breakpoints [6]. Resistance to macrolides, particularly erythromycin, was prevalent [10]. Cefotaxime resistance was also noted, reflecting reduced susceptibility to β-lactams in some isolates.

Statistical analysis 

Data analysis was conducted using Microsoft Excel (Microsoft® Corp., Redmond, WA) and R (version 4.2.0; R Development Core Team, Vienna, Austria) for statistical computations. The chi-square test was employed to examine associations between bacterial species (P. aeruginosa, A. baumannii, and K. pneumoniae) and their ESBL-producing status. The distribution of antimicrobial resistance among ESBL-producing and non-ESBL-producing bacteria was also analyzed. For categorical data with expected counts below five, Fisher’s exact test was used to ensure robust statistical inferences. All p-values were two-sided, and a p-value < 0.05 was considered statistically significant.

Ethical statement 

The study was conducted in full compliance with international ethical standards. It was approved by the Research Ethics Committee of the Medical Faculty at Tbilisi State Medical University in October 2022 (approval number: #1331). All patients provided informed consent for using their clinical data and samples in research, and assurances of confidentiality and privacy were maintained throughout the study.

## Results

**Figure 1:**
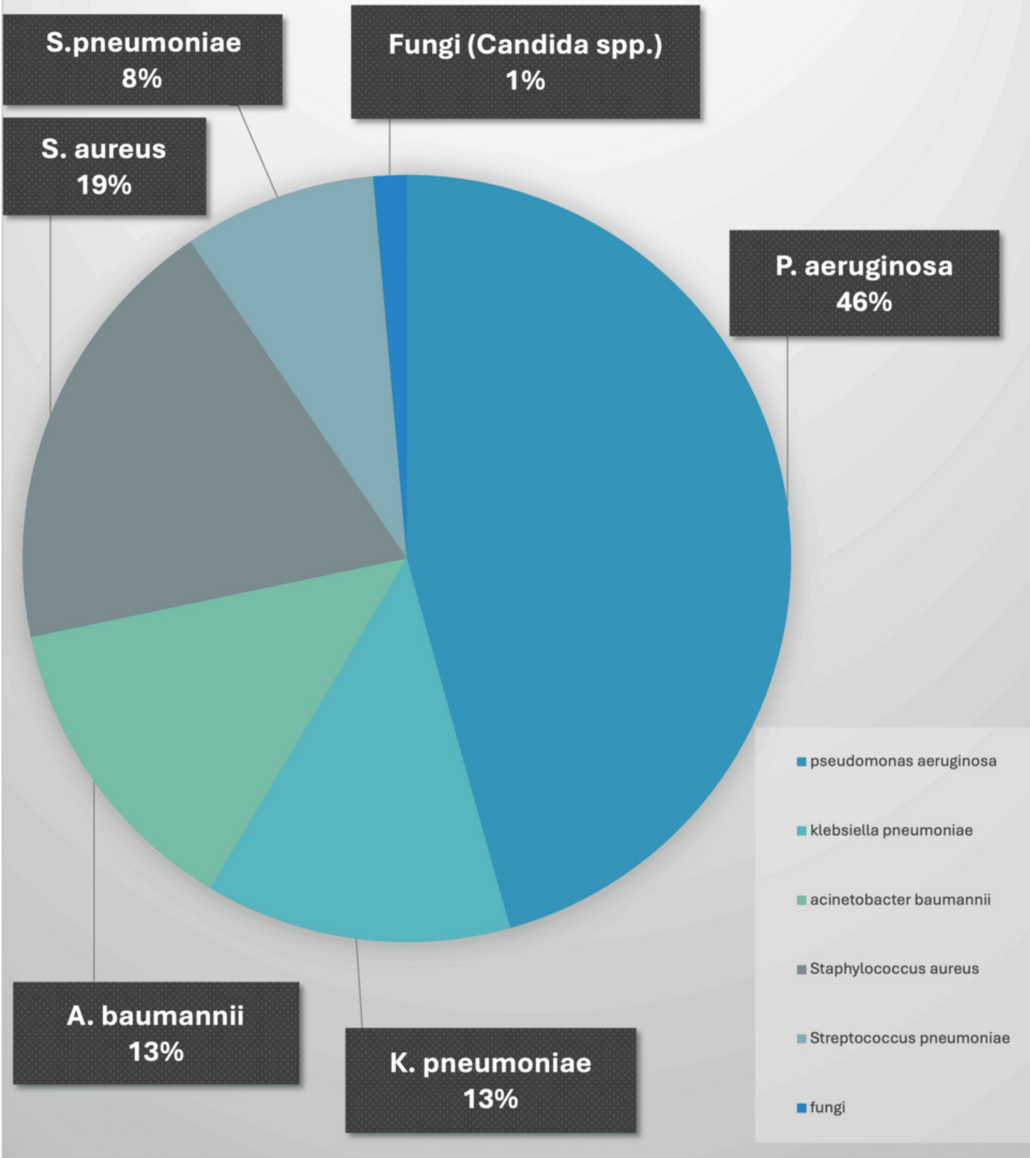
Percentage distribution of pathogens causing nosocomial pneumonia This pie chart illustrates the percentage distribution of pathogens identified in 357 samples from cases of nosocomial pneumonia. Pseudomonas aeruginosa accounts for the highest proportion (163/357, 46%), followed by Staphylococcus aureus (67/357, 19%), Acinetobacter baumannii (47/357, 13%), and Klebsiella pneumoniae (46/357, 13%), with lesser contributions from Streptococcus pneumoniae (29/357, 8%) and fungi (Candida spp.) (5/357, 1%). For a detailed breakdown of MRSA and MSSA proportions, please refer to Table 1.

The analysis of 357 pulmonary samples revealed a diverse spectrum of pathogens, with P. aeruginosa being the most frequently isolated organism, accounting for 46% of the total isolates. Other significant pathogens included K. pneumoniae and A. baumannii (13% each), S. aureus (19%), and S. pneumoniae (8%), with fungal isolates (Candida spp.) contributing to 1% of the cases (Figure 1).

Distribution of positive Gram-negative cultures

Among the 357 pulmonary samples analyzed, 256 (71.7%) were identified as positive for Gram-negative bacterial infections. The leading Gram-negative pathogens were P. aeruginosa (163/256 isolates, 63.7%), followed by A. baumannii (47/256 isolates, 18.4%) and K. pneumoniae (46/256 isolates, 17.9%). These findings highlight the predominant role of Gram-negative bacteria in nosocomial pneumonia cases in Georgian hospitals.

Distribution of positive Gram-positive cultures

In addition to the Gram-negative isolates, 96 samples (26.9%) were identified as positive for Gram-positive cocci. Among these, 67/96 samples (69.8%) were S. aureus isolates. MRSA represented 43 of these isolates, corresponding to 64.2% of the S. aureus cases. Furthermore, inducible clindamycin resistance (ICR) was observed in 38/67 isolates (56.7%), all of which were MRSA (Table 1).

**Table 1:** Distribution of microorganisms isolated from nosocomial pneumonia cases This table shows the distribution and prevalence of microorganisms isolated from nosocomial pneumonia cases, categorized into Gram-negative bacilli, Gram-positive cocci, and fungi. The Gram-negative bacilli include extended spectrum beta-lactamase (ESBL)-producing bacteria, such as Pseudomonas aeruginosa, Acinetobacter baumannii, and Klebsiella pneumoniae. The Gram-positive cocci section highlights Staphylococcus aureus isolates, including methicillin-resistant S. aureus (MRSA) and inducible clindamycin resistance, as well as Streptococcus pneumoniae with resistance to multiple antibiotics. The fungi section reports Candida species, which accounted for all fungal isolates in this study.

Gram-Negative Bacilli (N=256)		Gram-Positive Cocci (N=96)	Fungi (N=5)
ESBL Producers (201)			
Pseudomonas aeruginosa	123/201 (61.2%)	Staphylococcus aureus 67/96 (69.8%)	Candida species 5 (100%)
		Methicillin-resistant (MRSA) 43 (64.2%)	
		Inducible clindamycin resistance (all MRSA) 38 (56.7%)	
Acinetobacter baumannii	47/201 (23.4%)	Streptococcus pneumoniae 29/96 (30.2%)	
		Resistant to penicillin 29 (100%)	
		Resistant to vancomycin 2 (6.9%)	
Klebsiella pneumoniae	31/201 (15.4%)	Resistant to all beta-lactams 8 (27.6%)	
		Resistant to macrolides 24 (82.8%)	

Regarding S. pneumoniae, 29/96 isolates (30.2%) were identified. Among these, two isolates (6.9%) demonstrated resistance to vancomycin. Eight isolates (27.6%) exhibited resistance to all beta-lactam antibiotics. Macrolide resistance was observed in 24 isolates (82.8%), and all isolates were resistant to penicillins (Table 1).

Prevalence of ESBL-producing bacteria

**Figure 2:**
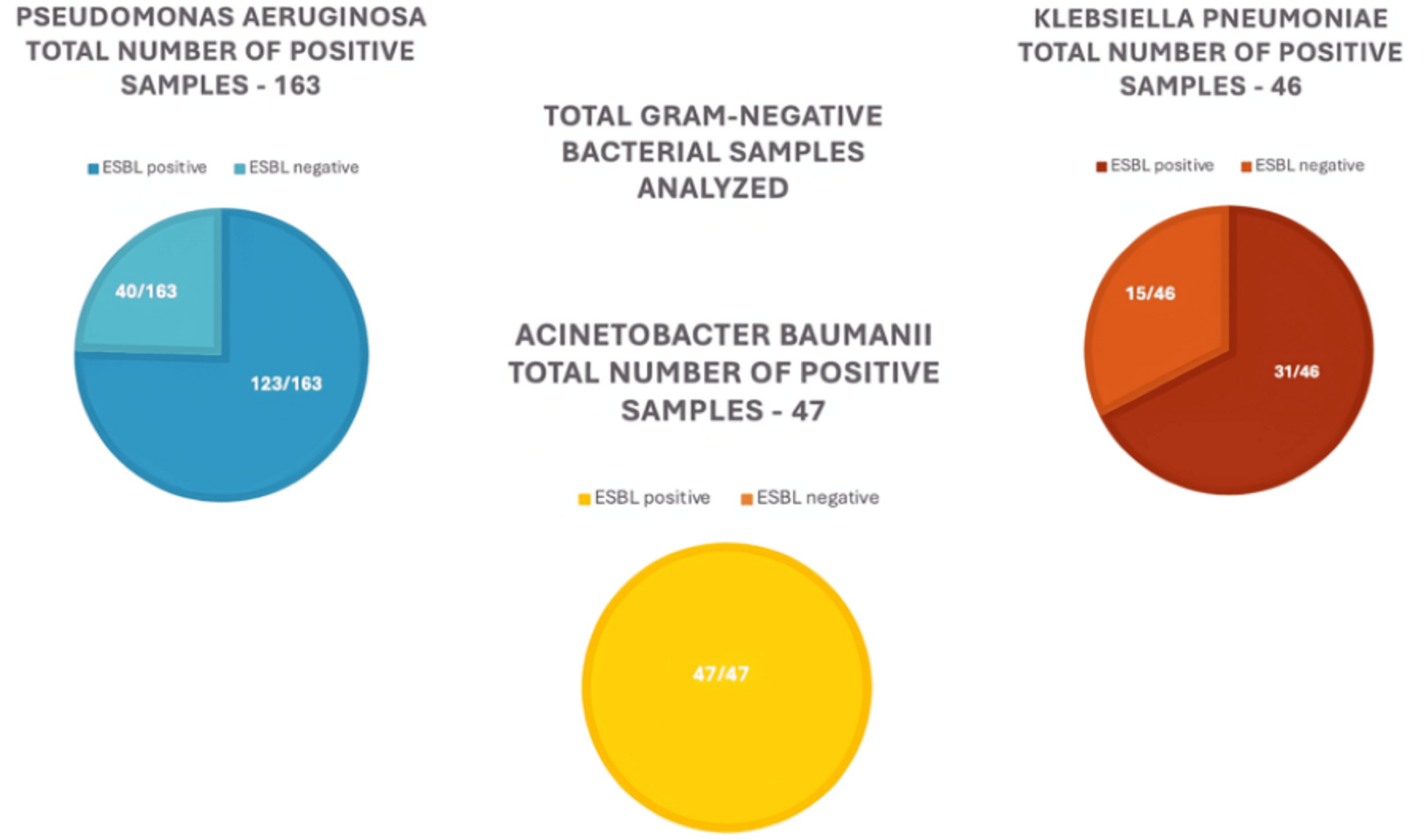
ESBL status in Gram-negative bacterial isolates from nosocomial pneumonia cases The figure depicts the distribution of ESBL-positive and ESBL-negative isolates among three key Gram-negative bacterial pathogens from nosocomial pneumonia cases (a total of 256 Gram-negative samples). Pseudomonas aeruginosa had 163 positive samples, with 123 (75.5%) being ESBL-positive and 40 (24.5%) ESBL-negative. Acinetobacter baumannii had 47 positive samples, all of which were ESBL-positive. Klebsiella pneumoniae had 46 positive samples, with 15 (32.6%) being ESBL-negative and 31 (67.4%) ESBL-negative.

Among the 256 Gram-negative isolates, 201 (78.5%) were identified as ESBL producers (Table 1). The species-specific prevalence of ESBL production varied, with all A. baumannii isolates (47/47) producing ESBLs. Out of all K. pneumoniae isolates, 31 out of 46 (67.4%) were identified as ESBL producers. Similarly, 75.5% of P. aeruginosa isolates (123 out of 163) and all A. baumannii isolates (47 out of 47) were also ESBL producers. A chi-square test revealed a statistically significant association between bacterial species and ESBL production (p = 0.002) (Figure 2).

Antibiotic resistance patterns

**Figure 3:**
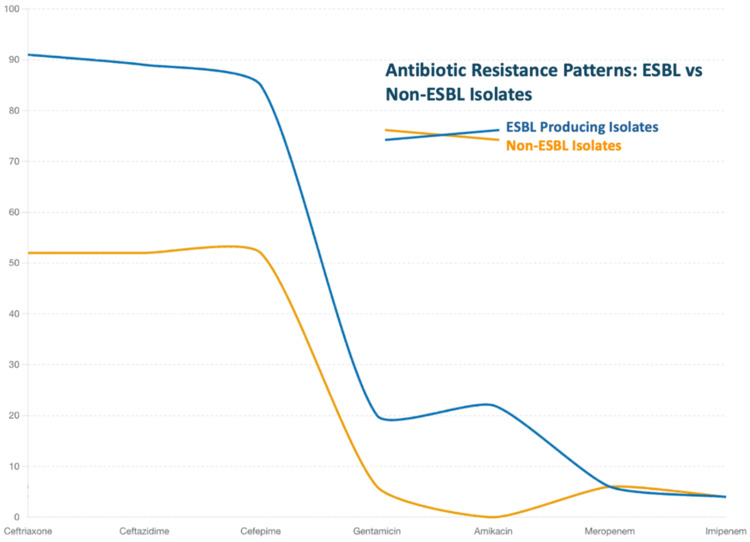
Antibiotic resistance patterns: ESBL vs non-ESBL isolates Blue line (ESBL-producing isolates): Beta-lactams: Resistance rates of 91% to ceftriaxone, 89% to ceftazidime, and 85% to cefepime. Carbapenems: Resistance rates of 6% to meropenem and 4% to imipenem. Aminoglycosides: 22% resistance to amikacin and 20% to gentamicin. Orange line (non-ESBL isolates): Beta-lactams: Resistance rate of 52%. Aminoglycosides: near 0% resistance to amikacin and 6% to gentamicin. Carbapenems: resistance rates of 6% to meropenem and 4% to imipenem.

Resistance to beta-lactams was significantly higher among ESBL-producing isolates. ESBL producers exhibited resistance rates of 91% to ceftriaxone (p < 0.001), 89% to ceftazidime (p = 0.004), and 85% to cefepime (p = 0.002). Non-ESBL-producing isolates demonstrated resistance to beta-lactams in 52% of cases (Figure 3).

Carbapenem resistance was low across all isolates, with meropenem and imipenem resistance rates of 6% and 4%, respectively. No significant difference in carbapenem susceptibility was observed between ESBL-producing and non-ESBL-producing isolates (p = 0.06) (Figure 3).

Aminoglycosides demonstrated high efficacy against most isolates. Amikacin resistance was observed in 22% of ESBL-producing isolates, while it was nearly 0% in non-ESBL isolates. Gentamicin resistance was 20% among ESBL producers and 6% among non-ESBL producers. A significant difference in gentamicin resistance was observed between ESBL and non-ESBL producers (p = 0.03) (Figure 3).

Fluoroquinolone resistance was pathogen-specific. A. baumannii demonstrated the highest ciprofloxacin resistance rate (88%, p < 0.001). Moderate ciprofloxacin resistance was observed in ESBL-producing K. pneumoniae isolates (65%, p = 0.02).

For Gram-positive cocci, MRSA isolates demonstrated high resistance to macrolides, with 74.4% of isolates showing resistance. Among S. pneumoniae isolates, significant resistance was observed against beta-lactams (27.6%) and macrolides (82.8%), while all isolates were resistant to penicillins.

Resistance trends by pathogen

**Figure 4:**
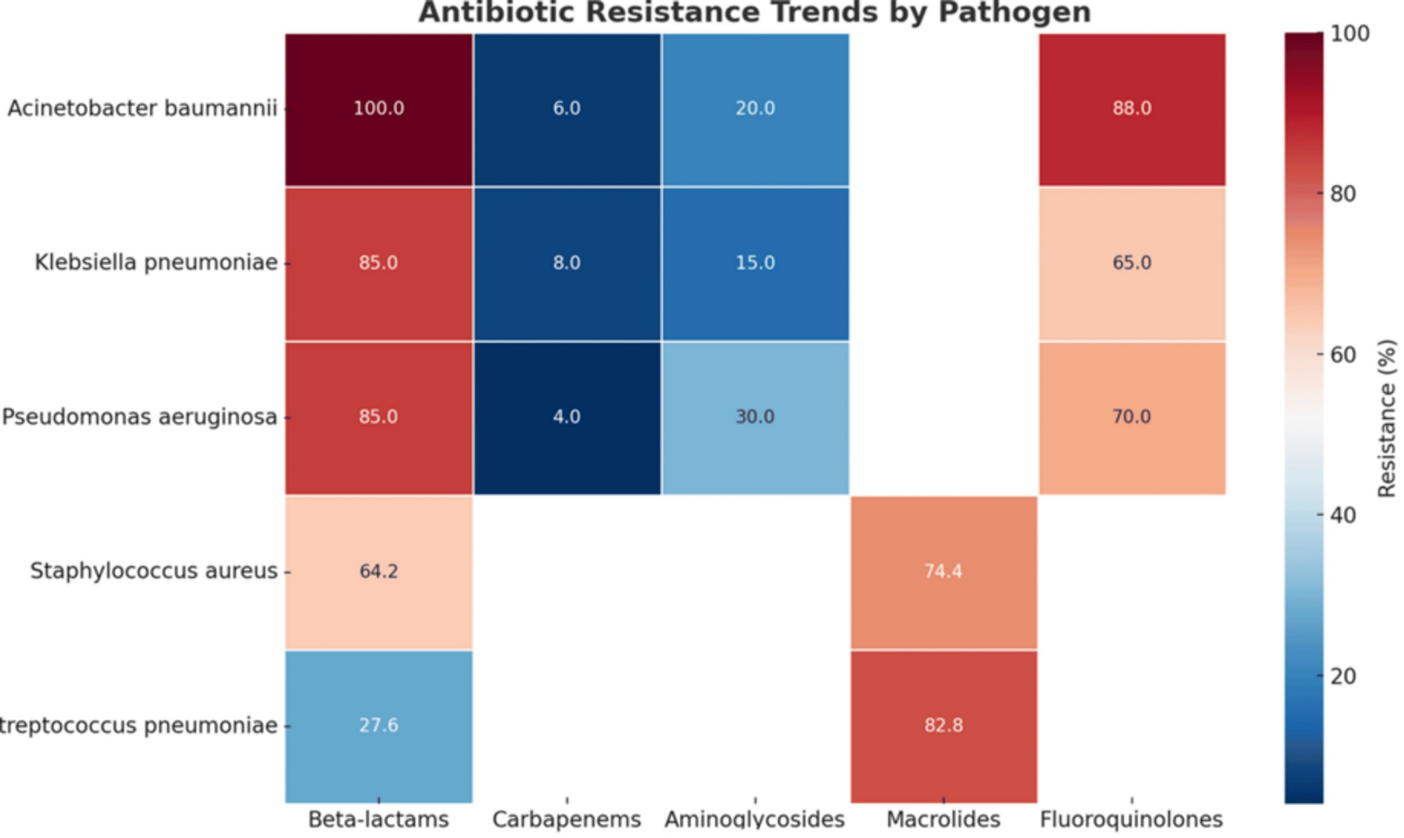
Antibiotic resistance trends by pathogen The Y-axis represents the bacterial species analyzed for resistance patterns, while the X-axis displays the categories of antibiotics tested, including beta-lactams, carbapenems, aminoglycosides, macrolides, and fluoroquinolones. The heatmap uses a color gradient where dark red indicates the highest resistance (approaching 100%) and dark blue indicates the lowest resistance (approaching 0%). The numeric values within the heatmap represent the percentage of isolates resistant to each antibiotic class for the corresponding pathogen, providing a comparative analysis of resistance rates across various pathogens and antibiotic classes.

A. baumannii exhibited universal ESBL production, 100% resistance to beta-lactams (p < 0.001), 20% resistance to aminoglycosides (p = 0.02), and the highest fluoroquinolone resistance at 88% (p < 0.001), while carbapenems remained effective in 94% of isolates (p = 0.01) (Figure 4).

P. aeruginosa demonstrated resistance rates of 85% to both ceftazidime and ceftriaxone among ESBL-producing isolates, compared to significantly lower resistance in non-ESBL producers (p = 0.03). Resistance to aminoglycosides was 30% (p = 0.02), while fluoroquinolone resistance was observed in 70% of isolates (p = 0.03). Carbapenem resistance remained low at 4% (p = 0.03). K. pneumoniae showed moderate carbapenem resistance (8%, p < 0.05) and fluoroquinolone resistance (65%, p = 0.02), while aminoglycoside resistance was 15% (p = 0.01), with 85% of isolates being susceptible to amikacin. Resistance to beta-lactams was 85% among ESBL-producing isolates (p = 0.001). S. aureus demonstrated a high prevalence of MRSA (64.2%, p = 0.001), and MRSA isolates exhibited high resistance to macrolides (74.4%, p = 0.001). S. pneumoniae showed significant resistance to beta-lactams (27.6%, p = 0.01) and macrolides (82.8%, p = 0.001), with all isolates being resistant to penicillins (p < 0.001) (Figure 4).

Statistical associations

A strong correlation was observed between ESBL production and resistance to cephalosporins across all Gram-negative species (chi-square test, p < 0.001). Fisher’s exact test revealed significant differences in gentamicin resistance (p = 0.03) and multidrug resistance rates for ESBL-producing K. pneumoniae isolates (p = 0.004). Resistance profiles for Gram-positive cocci revealed substantial beta-lactam and macrolide resistance, particularly in S. pneumoniae.

## Discussion

Nosocomial pneumonia represents a significant public health challenge, particularly in regions grappling with the increasing prevalence of MDR pathogens [1,2]. The findings of this study indicate a troubling dominance of Gram-negative bacteria, particularly P. aeruginosa (63.7%), A. baumannii (18.4%), and K. pneumoniae (17.9%). This aligns with global data, which consistently identifies these pathogens as major contributors to hospital-acquired infections [3-5,11]. P. aeruginosa, due to its intrinsic resistance mechanisms and adaptive capabilities, remains a leading cause of nosocomial pneumonia worldwide [8], while A. baumannii is particularly concerning for its role in outbreaks within intensive care units (ICUs) [8,9]​. In our opinion, the high prevalence of P. aeruginosa highlights the need for interventions addressing its resistance mechanisms. Similarly, A. baumannii outbreaks underline the importance of strict infection control in ICUs.

The prevalence of ESBL-producing strains among Gram-negative isolates (78.5%) underscores the critical challenge of antimicrobial resistance in healthcare settings [9,12]. ESBL production was universal in A. baumannii isolates and present in significant proportions of K. pneumoniae (67.4%) and P. aeruginosa (75.5%). These rates surpass those reported in studies from neighboring regions, where ESBL prevalence typically ranges from 50% to 70% [13,14]​. The association between ESBL production and resistance to third-generation cephalosporins was statistically significant (p < 0.002), reinforcing the role of ESBLs in reducing the efficacy of key antimicrobial agents [15,16]. In our view, the significantly higher prevalence of ESBL-producing strains in this study highlights the critical need to optimize current treatment protocols and prioritize preventive strategies to curb the spread of resistance.

Cephalosporin resistance among ESBL-producing isolates exceeded 85% in this study, consistent with findings from high-burden regions, including Southeast Asia and parts of the Middle East [17]. While carbapenems, including meropenem and imipenem, retained high efficacy (resistance rates of 4-6%), the potential for resistance emergence is a looming threat, as seen in studies reporting increasing carbapenemase-producing strains in similar settings [17-19]​. Aminoglycosides, particularly amikacin, offered a robust therapeutic option, with susceptibility exceeding 85% for P. aeruginosa and K. pneumoniae [19]. These findings align with global trends that favor aminoglycosides as a reliable component of combination therapies for MDR pathogens [20-22]. We believe that these findings highlight the necessity of preserving carbapenems as a last-resort treatment and prioritizing aminoglycosides as part of combination therapies to manage MDR pathogens effectively.

The study highlights A. baumannii as a pathogen of critical concern, demonstrating universal resistance to beta-lactams and high resistance to ciprofloxacin (88%). These resistance rates reflect the pathogen’s ability to acquire resistance genes rapidly and survive in hospital environments [11,13]. This is comparable to findings in East Asia and Southern Europe, where A. baumannii outbreaks are frequently linked to carbapenem resistance [14,17]. In our opinion, this underscores the importance of early detection and strict infection control measures to prevent the spread of A. baumannii.

In Gram-positive cocci, MRSA accounted for 64.2% of S. aureus isolates, with macrolide resistance rates reaching 74.4%. These figures echo resistance profiles in resource-limited settings, where infection control measures may be less stringent [23,24]. Similarly, S. pneumoniae exhibited significant resistance to beta-lactams (27.6%) and macrolides (82.8%), posing challenges for conventional therapies [24]. Resistance to penicillins among all S. pneumoniae isolates underscores the pressing need for alternative treatment strategies and enhanced vaccine coverage to mitigate its clinical impact [25]​. In our assessment, these findings underscore the urgency of enhancing infection control measures and expanding vaccine coverage in high-risk populations to mitigate the burden of drug-resistant Gram-positive pathogens.

Age-related resistance trends are particularly noteworthy, with older adults demonstrating higher resistance rates to carbapenems and fluoroquinolones. This aligns with global observations that identify advanced age as a risk factor for MDR infections due to cumulative antibiotic exposure, comorbidities, and prolonged hospital stays [4,26,27]. By contrast, younger patients showed greater susceptibility to standard therapies, underscoring the need for age-specific treatment approaches [28]​. These findings suggest the necessity of carefully assessing risk factors, such as comorbidities and prior antibiotic use, before selecting appropriate antibiotic therapies for different age groups. Tailored approaches can optimize treatment outcomes and reduce the risk of resistance development.

Efforts to combat nosocomial pneumonia require a multi-pronged approach. The observed resistance patterns necessitate stringent antibiotic stewardship programs (ASPs) aimed at optimizing antimicrobial use and curbing the overprescription of cephalosporins [11,20]. The integration of infection control measures, including active surveillance, hand hygiene compliance, and environmental decontamination, is critical to reducing the transmission of MDR pathogens [4,17]. Empirical treatment strategies should be informed by local antibiograms to ensure targeted and effective therapy [29]. We suggest that addressing nosocomial pneumonia demands a comprehensive approach. Implementing effective ASPs to optimize antimicrobial use and limit cephalosporin overprescription is essential. Strengthening infection control measures, including active surveillance, strict hand hygiene, and environmental decontamination, can greatly mitigate the spread of MDR pathogens. Additionally, customizing empirical treatments based on local antibiograms will improve the accuracy and efficacy of therapeutic strategies.

Limitations

This study focused exclusively on culturable bacteria, potentially underestimating the contribution of fastidious or anaerobic pathogens to nosocomial pneumonia. Additionally, reliance on phenotypic methods for ESBL detection may have excluded non-classical resistance mechanisms. Molecular diagnostic tools and whole-genome sequencing could provide a more comprehensive understanding of resistance determinants in future research.

## Conclusions

The findings emphasize the critical burden of MDR pathogens in nosocomial pneumonia, with high prevalence rates of ESBL-producing P. aeruginosa, A. baumannii, and K. pneumoniae. The limited efficacy of cephalosporins and rising resistance to fluoroquinolones necessitate reliance on carbapenems and aminoglycosides as primary treatment options. However, the looming threat of resistance to these agents underscores the urgency of implementing robust ASPs and infection control practices. These findings serve as a foundation for evidence-based interventions to mitigate antimicrobial resistance in Georgian healthcare settings.
